# The HBV Specially-Related Long Noncoding RNA HBV-SRL Involved in the Pathogenesis of Hepatocellular Carcinoma

**DOI:** 10.1155/2022/9034105

**Published:** 2022-07-08

**Authors:** Cunzhen Zhang, Lei Lu, Haibei Xin, Minfeng Zhang, Zhiwen Ding, Qiaomei Li, Kuang Chen, Minggen Hu, Shupeng Liu, Nan Li

**Affiliations:** ^1^Department of Hepatic Surgery I (Ward I) Shanghai Eastern Hepatobiliary Surgery Hospital, Second Military Medical University, Shanghai, China; ^2^Changhai Hospital, Second Military Medical University, Shanghai, China; ^3^Faculty of Hepato-Pancreato-Biliary Surgery, Chinese PLA General Hospital, Beijing, China; ^4^Department of Obstetrics and Gynecology, Shanghai Tenth People's Hospital, Tongji University, Shanghai, China

## Abstract

Hepatitis B virus (HBV) is one of the major risk factors for HCC (hepatocellular carcinoma) occurrence with a diverse role in the pathogenesis of HCC. More works need to be performed to elucidate a more thorough understanding of the molecular mechanisms involving in HBV-induced HCC, although some mechanisms such as genome integration have been reported. In the present study, aberrantly expressed lncRNAs were identified between HCC tumor tissues with or without HBV infection. Among these molecules, HBV specially-related long noncoding RNA (HBV-SRL) was further found to correlate with poor prognosis and a shorter overall survival time in HCC patients with HBV infection. Additionally, HBV-SRL was found function as oncogene by upregulating the NF-*κ*B2 expression. These data suggest that HBV infection altered gene expression pattern in liver cells which contributed to HBV-related HCC development, and HBV-SRL may serve as a new molecular marker or potential therapeutic target of HBV-related HCC.

## 1. Introduction

Hepatitis B virus (HBV) infection is one of the major risk factors for HCC (hepatocellular carcinoma) occurrence [1]. More than half of all patients with HCC are etiologically linked to an infection [2]. Improvement in the prognosis of this disease via *anti*-HBV therapy has highlighted the crucial role of HBV in HCC pathogenesis and progression [3]. The integration of HBV DNA into the genome of hepatocytes was reported to be the main reason of HBV-related HCC (HBV-HCC) development due to its role in promoting genomic instability and mutagenesis [4]. Viral proteins such as HBX contributed to HBV-HCC via signaling through Akt or NF-*κ*B pathways, modulating DNA methylation and histone modification patterns, among other roles [5]. Persistent inflammation and antiviral response induced by HBV infection increased host susceptibility to malignant transformation [6]. Taken together, these findings suggest a diverse role of HBV in the pathogenesis of HCC. However, more work needs to be performed to elucidate a more thorough understanding of the molecular mechanisms of HBV-induced HCC.

Long noncoding RNAs (lncRNAs), a novel type of noncoding RNA with pivotal roles in epigenetic regulation, were found to function as regulators of viral replication or antiviral response [7, 8]. Both viral lncRNAs and cellular lncRNAs induced by infection were found in infected cells [9]. Viral lncRNAs promoted viral replication via autonomous replication [10] and decreased the antiviral response by transcriptionally regulating the viral and host genomes [11] or transcriptomes alike [12]. Cellular lncRNAs induced by infection were mainly found to participate in the antiviral response. Cellular lncRNAs such as NEAT1 (nuclear enriched abundant transcript 1) and BISPR (bone marrow stromal antigen 2 IFN-stimulated positive regulator) were found to activate the *anti*viral response by modulating *anti*virus factors [13], while lncRNAs such as lncRNA IL7R and THRIL (TNF-alpha and heterogeneous nuclear ribonucleoprotein *L*-related immunoregulatory LncRNA) counteracted the *anti*viral response by negatively controlling the IFN pathway [14–16]. Aside from their role in viral infection or suppression, lncRNAs have also been shown to be involved in diverse biological cellular processes including tumorigenesis [17]. Aberrantly expressed lncRNAs, such as H19 and MVIH, had been identified in multiple tumors including HCC [18]. H19 in liver cancer cells induces drug resistance through regulation of multidrug resistance 1 (MDR1) promoter methylation and suppressed metastasis via a miR-220 dependent pathway [19]. MVIH (microvascular invasion in HCC) promoted tumor growth and intrahepatic metastasis by activating angiogenesis [20]. In addition, our previous study found that ICR (ICAM-1-related lncRNA), which is expressed in liver cancer stem cells (CSC), regulated CSC properties of ICAM-1^+^ HCC cells and promoted tumor cell migration [21]. These findings highlight the crucial roles of lncRNA in the pathogenesis and progression of HCC. Thus, it is possible that lncRNAs impact HBV-induced HCC. While lncRNAs expressed in HBV-HCC such as highly upregulated in liver cancer (HULC) and HBX-long interspersed nuclear elements 1 (HBX-LINE1) have been identified, additional studies are required to fully characterize the effect of lncRNAs in HBV-induced HCC.

In the present study, we analyzed the expression profile of lncRNA and mRNA in HBV-related and unrelated HCC tissues using cDNA microarray and found differently expressed lncRNAs and mRNAs between these tissues. One of the differentially expressed lncRNAs, termed as HBV specially-related long noncoding RNA (HBV-SRL), was found to be upregulated in HBV-induced HCC. HBV-SRL correlated with the poor prognosis and reduced survival of HCC patients with HBV infection. Functionally, HBV-SRL promoted tumor cell proliferation by upregulating NF-*k*B2 via activating NF-*k*B2 transcription. Our findings suggest that HBV-SRL may represent a new molecular marker and potential therapeutic target of HBV-induced HCC.

## 2. Materials and Methods

### 2.1. Patients and Follow-Up

HCC patients with or without HBV (*n* = 232) who underwent radical cancer resection at Eastern Hepatobiliary Surgery Hospital (EHBH) were recruited for microarray analysis and validation, respectively, in the present study. The clinical characteristics of the patients are listed in Supplementary Table 1. The study was approved by the Institutional Review Board of the EHBH. Other details of patient information are listed in Supplementary Materials.

### 2.2. Statistical Analysis

Student's *t*-tests were used to compare two groups unless otherwise indicated (*χ*^2^ test). Categorical data were analyzed using the Fisher exact test, and quantitative variables were analyzed using the *t* tests or Pearson's correlation test. Survival was calculated with the log-rank test. The Cox regression model was used to perform multivariate analysis. *P* < 0.05 was considered statistically significant.

Detail of other materials and methods is listed in Supplementary Materials.

## 3. Results

### 3.1. HBV-SRL Was Higher in Hepatocellular Carcinoma with HBV

To identify lncRNAs associated with HBV-HCC, we collected HCC tumor tissues with (Group 1, *n* = 5, HBV^+^) or without HBV (Group 2, *n* = 5, HBV^−^) infection and analyzed lncRNA expression profiles in these tissues using cDNA microarrays.

**Figure 1:**
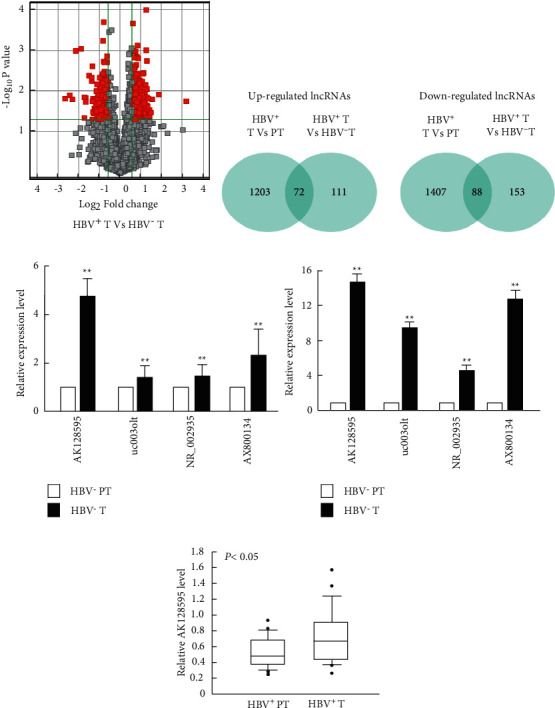
Differentially expressed lncRNAs between HCC tumors with or without HBV infection. (a) Microarray analysis found differential expression of lncRNA between tumor tissues with HBV (HBV^+^T) and that without HBV (HBV^−^T). (b) Bioinformatic analysis of differently expressed lncRNAs (fold change > 2) between tumor tissues with HBV (HBV^+^T) and that without HBV (HBV^−^T) or tumor tissues and corresponding parenchyma tumor tissues (PT). (c) Real-time PCR analysis of 4-candidate lncRNAs expression in tumor tissues (T) and corresponding parenchyma tumor tissues (PT) without (A) or with HBV (B). (d) Real-time PCR analysis of lncRNA AK128595 expression in tumor tissues (HBV^+^T) and corresponding parenchyma tumor tissues (HBV^+^PT) from patients with HBV (*n* = 30). The error bars represent the standard deviation (SD) of data obtained in at least three independent experiments, ^*∗∗*^*P* < 0.01.

Differentially expressed lncRNAs profiles were identified between tumor tissues (T) and the corresponding parenchyma tumor tissues (PT) in both groups (Supplementary Figure S1), indicating the involvement of lncRNAs in HCC development. To find out HBV specific-related lncRNAs, differentially expressed lncRNAs between HBV^+^ and HBV^−^ tumors were also investigated. 183 upregulated lncRNAs and 241 downregulated lncRNAs were identified to be differentially expressed between HBV^+^ and HBV^−^ tumors (Figures 1(a) and 1(b)). Among these lncRNAs, 72 lncRNAs were found upregulated in HBV^+^ tumors compared with the corresponding HBV^+^ PTs and 88 lncRNAs were found downregulated (Figure 1(b)). Based on these findings, four upregulated lncRNAs with a 6-fold greater expression in HBV + T Vs and HBV + PT and 2.5-fold greater in HBV ^+^ T Vs and HBV^−^T were selected as candidate (Supplementary Table 2). We validated our microarray results using real-time PCR, highlighting the elevated expression of our candidate lncRNAs in T versus PT in both HBV^−^ (Figure 1(c)) and HBV^+^ patients (Figure 1(c)). AK128595 was selected for further analysis due to its greatest increase. Then, the AK128595 expression was verified using a validation cohort of 30 HBV^+^ tumors, and the result showed higher level of AK128595 in HBV^+^T compared with HBV^+^ PT (Figure 1(d)). In the present study, AK128595 was identified as HBV-related long noncoding RNA (HBV-SRL).

### 3.2. HBV-SRL Produced No Protein HBV-SRL

**Figure 2:**
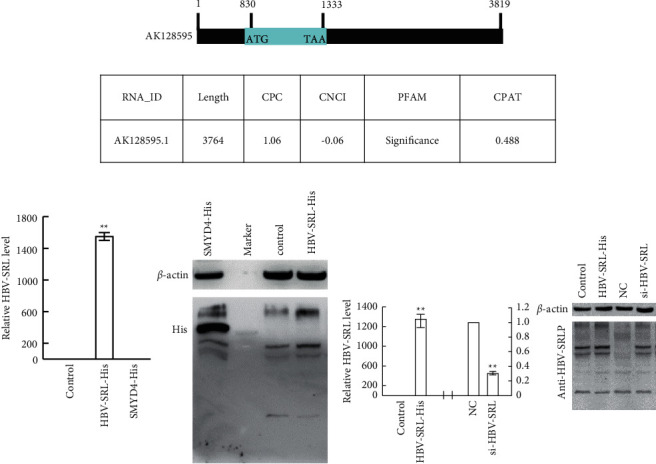
Investigation of HBV-SRL coding capacity. (a) Diagram of HBV-SRL transcript. Potential open reading frame (ORF) ranged from 830 to 1333. (b) Bioinformatic analysis of coding capacity of HBV-SRL using CPC, CNCI, PFAM, and CPAT. CPC, coding potential calculator; CNCI, coding-noncoding index; CPAT, coding potential assessment tool. Transcript with CPC score < 0, CNCI score < 0, PFAM = nonsignificant, and CPAT <0.364 was a potential lncRNA. (c) Real-time PCR analysis of HBV-SRL (A) and western blot analysis of His expression (B) in CSQT-2 cells transfected with plasmid pAdeno-His (Control), pAdeno-HBV-SRL-His (HBV-SRL-His), or pSMYD4-His (SMYD4- His). (d) Real-time PCR analysis of HBV-SRL (A) and western blot analysis by anti-HBV-SRLP antibody (B) using CSQT-2 cells transfected with plasmid pAdeno-His (Control), pAdeno-HBV-SRL-His (HBV-SRL-His), or siRNAs targeting HBV-SRL (si-HBV-SRL). The error bars represent the standard deviation (SD) of data obtained in at least three independent experiments, ^*∗∗*^*P* < 0.01.

An evaluation of the HBV-SRL sequence identified an open reading frame (ORF) with 504 nucleotides in length, suggestive of a potential protein product (Figure 2(a)). Bioinformatic analysis (CPC, CNCI, and PFAM) also found the coding potential of HBV-SRL (Figure 2(b)). To evaluate these findings, a pAdeno-HBV-SRL-His plasmid expressing HBV-SRL with polyhistidine (6XHis) insertion into the ORF just before the stop codon was transfected into CSQT-2 cells (Supplementary Figure S2). After validating enhanced HBV-SRL expression (Figure 2(c)), His antibody was used to detect whether His-tagged protein was produced via western blot. No specific signal was observed in cells transfected with pAdeno-His (Control) or pAdeno-HBV-SRL-His (HBV-SRL-His), while an enhanced signal was observed in cells transfected with pSMYD4-His (SMYD4-His) (Figure 2(c)), indicating that no His-tagged protein was produced by pAdeno-HBV-SRL-His. Moreover, antibodies against the potential HBV-SRL protein (*anti*-HBV-SRLP) were produced using the synthesized peptide. Following HBV-SRL upregulation by pAdeno-HBV-SRL-His transfection and subsequent downregulation by siRNA transfection in CSQT-2 cells (Figure 2(d)), *anti*-HBV-SRLP was used to detect protein changes. No significant differences in western blot analysis were observed despite changes in HBV-SRL mRNA (Figure 2(d)). The similar results were also observed in Hep3B cells (Supplementary Figure S3). These data indicated that no protein can be produced by HBV-SRL, and HBV-SRL was a noncoding RNA.

### 3.3. HBV-SRL Functions as an Oncogene by Upregulating the NF-*κ*B2 Expression

**Figure 3:**
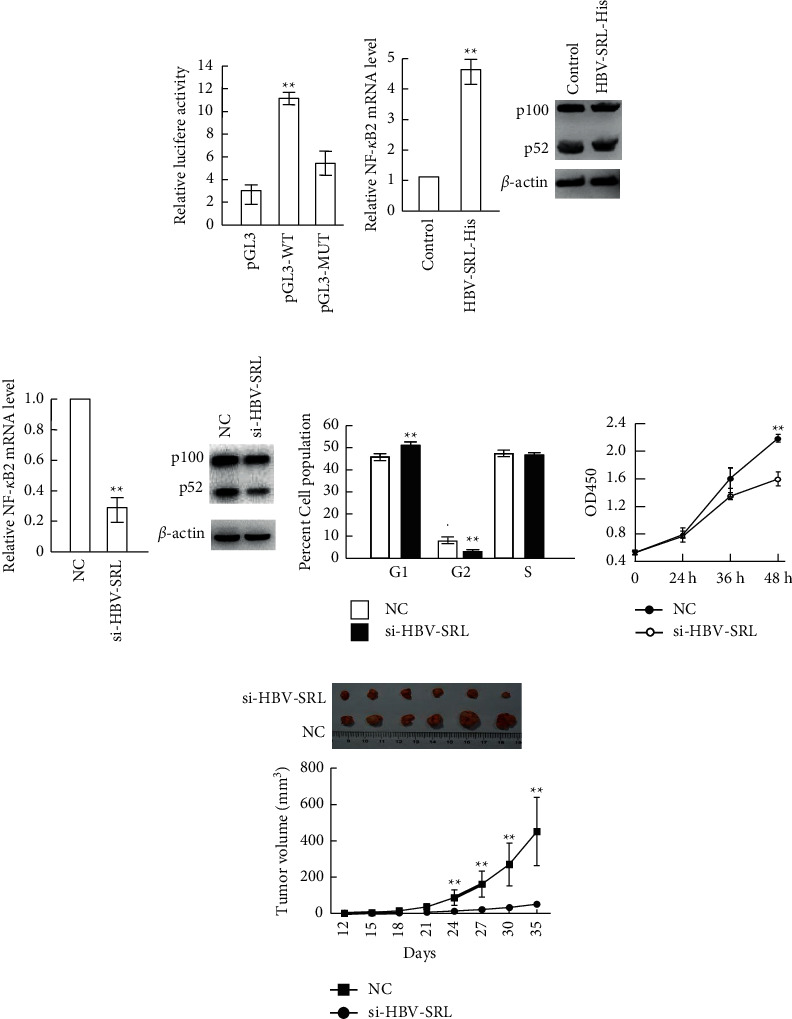
Regulation of NF-*k*B2 and tumor cells growth by HBV-SRL. (a) Luciferase analysis using CSQT-2 cells transfected with pAdeno-HBV-SRL-His and pGL3-*NF-κ*B2 promoter-wild type (pGL3-WT), pGL3-*NF-κB2* promoter-mutant (pGL3-MUT), or pGL3-enhancer (pGL3). (b) Real-time PCR (A) and western blot (B) analysis of NF-*κ*B2 in CSQT-2 cells transfected with plasmid pAdeno-His (Control) and pAdeno-HBV-SRL-His (HBV-SRL-His). (c) Real-time PCR (A) and western blot (B) analysis of NF-*κ*B2 in CSQT-2 cells transfected with siRNAs targeting HBV-SRL (si-HBV-SRL) and NC control (NC). (d) Cell cycle (A) or cell growth (B), CSQT-2 cells transfected with siRNAs targeting HBV-SRL (si-HBV-SRL) and NC control (NC) analyzed by flow cytometry and CCK-8, respectively. (e) Image (upper) and tumor growth curve (lower) of tumor tissues from mice treated with siRNAs targeting HBV-SRL (si-HBV-SRL) or negative control (NC). *N* = 6 each group. The error bars represent the standard deviation (SD) of data obtained in at least three independent experiments, ^*∗∗*^*P* < 0.01.

Having documented that HBV-SRL does not produce a protein product, we investigated how the HBV-SRL expression affects tumor cells. To this end, we aligned the HBV-SRL sequence with the gene promoters (upstream 1.5 kb) using Blat to find out promoter regions in which HBV-SRL can bind, since lncRNAs was previously reported to function as the transcription factor [22]. RELB and NF-*κ*B2 were retrieved, while the RELB promoter had 324 bp nucleotides similar to HBV-SRL and the NF-*κ*B2 promoter had 238 bp nucleotides complemented with HBV-SRL (Supplementary Table 3). The effects of HBV-SRL on NF-*κ*B2 were further investigated to functionally validate whether HBV-SRL binds to the NF-*κ*B2 promoter. The dual-luciferase reporter assay was performed to investigate whether HBV-SRL binds to the NF-*κ*B2 promoter. After HBV-SRL was upregulated by pAdeno-HBV-SRL-His transfection into CSQT-2 cells, pGL3-*NF-κB2* promoter-wild type (pGL3-WT) or pGL3-NF-*κ*B2 promoter-mutant (pGL3-MUT) with the complementary sequence deleted was transfected. The dual-luciferase reporter assay showed an higher luciferase activity in cells transfected with pGL3-WT compared with those transfected with the pGL3-enhancer (pGL3) (Figure 3(a)), and no significant change in luciferase activity was observed in CSQT-2 cells transfected with pGL3-MUT, suggesting that HBV-SRL binds to the NF-*κ*B2 promoter via sequence complement. To verify whether HBV-SRL regulates the NF-*κ*B2 expression, NF-*κ*B2 mRNA and protein were measured after upregulation or downregulation of HBV-SRL. Higher levels of NF-*κ*B2, at the both mRNA and protein levels, were observed when HBV-SRL was upregulated in both CSQT-2 and Hep3B cells (Figure 3(b) and Supplementary Figure S4(a)). Conversely, reduced NF-*κ*B2 mRNA and protein were observed when HBV-SRL was downregulated (Figure 3(c) and Supplementary Figure S4(b)), suggesting that HBV-SRL regulated the expression of NF-*κ*B2. As NF-*κ*B2 was reported to regulate tumor cell growth [23, 24], cell cycle and proliferation capacity of tumor cells were assessed following HBV-SRL downregulation. When HBV-SRL was downregulated by siRNAs (si-HBV-SRL) in CSQT-2 cells, tumor cells displayed higher percentage of cells in G1 phase and lower percentage of cells in G2 phase (Figure 3(d) and Supplementary Figure S5(a)). This alteration of cell cycle induced by HBV-SRL downregulation was also observed in Hep3B cells (Supplementary Figures S5(b) and S5(c)), indicating that HBV-SRL affected cell cycle. Moreover, CSQT-2 cell growth was slowed down by HBV-SRL downregulation (Figure 3(d)). In addition, the mice model was established by injecting Hep3B cells into mice subcutaneously to investigate whether HBV-SRL downregulation inhibited tumor growth *in vivo*. When tumor nodes appeared, si-HBV-SRL or NC was administrated in situ twice a week for four weeks. During this period, the tumor size was measured every three days. Five weeks later, all the mice were sacrificed and tumor tissues were weighted and sent for further analysis. Tumors treated with si-HBV-SRL were much smaller in volume than those treated with NC (*N* = 6 for each group) (Figure 3(e)). Tumor growth curve showed that growth of tumors administrated by si-HBV-SRL was much slower than that of control tumors (Figure 3(e)), indicating that si-HBV-SRL administration slowed down tumor growth in mice. The results show that HBV-SRL can regulate tumor cell cycle and growth via NF-*κ*B2 regulation.

### 3.4. HBV-SRL/NF-*κ*B2 Is Differentially Expressed in Tumors Compared to Paired Liver in Different HBV DNA Levels

**Figure 4:**
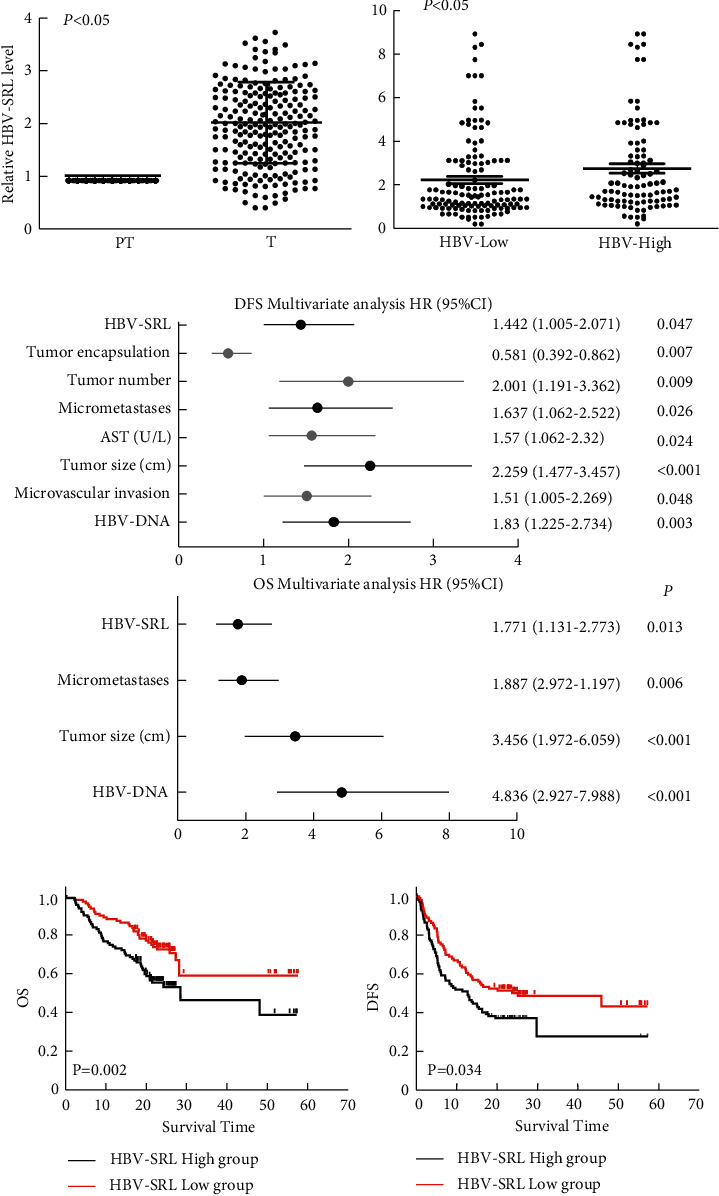
Correlation of the HBV-SRL expression with prognosis of HCC patients with HBV infection. (a) Real-time PCR analysis of HBV-SRL expression in tumor tissues (T) and corresponding parenchyma tumor tissues (PT) from 222 HCC patients with HBV. The error bars represent the standard deviation (SD) of data obtained in at least three independent experiments, ^*∗∗*^*P* < 0.01. (b) Expression of HBV-SRL in tumor tissues with high (≥104copy/ml, HBV- High) or low (<104 copy/ml, HBV-Low) HBV DNA level. (c) Association of HBV-SRL with disease-free survival (DFS) or overall survival (OS) of HCC patients with HBV infection analyzed by multivariate Cox hazards model. (d) Correlations of HBV-SRL with DFS and OS of 222 HCC patients analyzed by Kaplan–Meier's analyses.

Having demonstrated that HBV-SRL upregulated the NF-*κ*B2 expression and was involved in cell migration *in vitro*, we evaluated the relationship between the HBV-SRL and NF-*κ*B2 expression and recurrence in HCC patients with different level of HBV DNA. For this purpose, 222 HCC patients were analyzed as a validation cohort. Real-time PCR was used to measure the expression of HBV-SRL and NF-*κ*B2 in tumors (*T*) and paired liver tissues (PT). Both the expression of HBV-SRL and NF-*κ*B2 were higher in tumor tissues than in paired liver tissues (Figure 4(a) and Supplementary Figure S6(a)). According to the results of auxiliary examination (HBV DNA quantitative test), 222 HCC patients were divided into two groups: high HBV DNA level (HBV DNA ≥ 10^4^copy/ml) (*n* = 95) and low HBV DNA level (HBV DNA < 10^4^copy/ml) (*n* = 127). We then compared the relative expression level of HBV-SRL (T Vs PT) between the two groups. Higher level of HBV-SRL was observed in the high HBV DNA level group (HBV-high) (Figure 4(b)). We then analyzed the relationship between the HBV-SRL expression and clinicopathologic features in HCC patients and identified association between the HBV-SRL expression and absent tumor encapsulation (*P*=0.044) (Table 1). Univariate analysis found HBV-SRL as well as NF-*κ*B2 and HBV.

DNA was associated with DFS and OS (Table 2). Further, multivariate Cox hazards analysis found the association of HBV-SRL with prognosis in HBV-related HCC patients (Figure 4(c)). The median DFS and OS were 20.13 months and 22.90 months, respectively, in patients with low HBV-SRL expression. These findings were significantly longer than those observed for patients with a high HBV-SRL expression (12.63 and 20.20 months, respectively; both *P* < 0.001). Kaplan–Meier analysis demonstrated that patients with low HBV-SRL expression had better prognosis than those with a high HBV-SRL expression (Figure 4(d)). The NF-*κ*B2 expression was also associated with DFS and OS (Supplementary Figures 6(b) and 6(c)). The results suggested that both the high expressions of HBV-SRL and NF-*κ*B2 correlate with the poor prognosis of HCC patients with HBV infection.

## 4. Discussion

In the present study, we performed a transcriptional analysis of lncRNA expression in tumor tissues with and without HBV infection. Among differentially expressed lncRNAs, HBV-SRL was investigated due to *in silico* predictions of interactions with the NF-*κ*B2 promoter and subsequent *in vitro* studies validating the effects of HBV-SRL on the NF-*κ*B2 expression. Additionally, the HBV-SRL level was found to positively correlate with HBV DNA status and associate with poor prognosis of HCC patients with HBV infection. The results suggest that HBV infection induced the aberrant expression of lncRNAs that contributed to HCC development.

Nowadays, several studies have reported that HBV infection altered the expression of noncoding RNAs such as miRNAs and lncRNAs in liver cells. Our previous study and others found aberrant miRNA expression in the liver of HBx transgenic mice contributed to the HCC development [25, 26]. Similarly, microarray analysis of HBV-related HCC and normal liver tissues showed an altered lncRNA expression profile in tumor tissues, further indicating the crucial role of lncRNA in HBV-related HCC [27, 28]. LncRNAs such as highly upregulated LncRNAs in liver cancer (HULC) and HBX-long interspersed nuclear elements 1 (HBX-LINE1) have been also reported to contribute to HBV-induced HCC development via different ways [29]. By comparing with gene expression profiles from HBV^+^ HCC tumor tissues and HBV-HCC tumor tissues, we identified a unique gene expression profile in HBV^+^ HCC tumor tissues, which included lncRNAs. Moreover, our analysis highlighted the association of genes involved in tumor development and recurrence with aberrantly expressed lncRNAs induced by HBV infection (data not shown). These findings suggested a crucial and complex role by which HBV infection lead to the development of HCC. Besides, different gene expression profiles were also found between HBV-HCC tumors and the corresponding parenchyma tumor tissues, indicating that there many genes contributed to tumor development without HBV affection.

HBV infection is one of the most important factors associated with the poor prognosis of HBV-related HCC. In China, more than 90% of patients are found to have advanced HCC with HBV when the liver cancer is first diagnosed [30]. Although TACE, radiotherapy, and symptomatic treatment were used, hepatectomy still remains the most effective treatment [31]. However, not all patients have a bad prognosis due to the high HBV DNA level. Our results showed that HBV-SRL may be a novel predictor associated with malignant features of HBV-related HCC and is correlated with patients' prognosis. Moreover, HBV-SRL was found to associate with absent tumor encapsulation which suggested that HBV-SRL may be correlated with malignant tumors progression. HCC patients with high levels of HBV-SRL in parenchyma tumor should be monitored at increased intervals to prevent tumor metastasis or recurrence.

Long noncoding RNAs function as key regulators in multiple cellular processes including tumorigenesis via numerous molecular mechanisms [32]. In the present study, we found that lncRNA HBV-SRL functions as an oncogene regulating NF-*κ*B2 expression via binding to the NF*-κ*B2 promoter. HBV-SRL acts as a transcription factor with a detailed understanding of the molecular mechanism of its actions remains to be elucidated. Moreover, we found the slowdown of tumor cell growth by HBV-SRL downregulation via affecting cell cycle. However, HBV-SRL may have roles in other biological processes such as inflammation and immune response due to its regulation of NF*-κ*B2 which have crucial roles in these processes [33].

Additionally, the coding capacity of HBV-SRL was investigated by both bioinformatic analysis and *in vitro* experiments. No protein produced by pAdeno-HBV-SRL-His was observed in pAdeno-HBV-SRL-His transfected tumor cells although three out of four bioinformatic analysis tools indicated that HBV-SRL could have protein coding ability (Figure 2). It has been reported that several RNA transcripts annotated as lncRNAs produce micropeptides with biological functions [34, 35]. Thus, evaluating the protein coding capacity using experiments is necessary.

In conclusion, we describe a novel HBV-related lncRNA HBV-SRL that contributes to the pathogenesis of HCC in patients with HBV infection. High tumor HBV-SRL expression level was associated with higher rates of recurrence and poor prognosis after hepatectomy in HBV-related HCC, suggesting that HBV-SRL may serve as a new diagnostic marker for tumor recurrence and a potential target for HBV-related oncogenes inhibition.

## Figures and Tables

**Table 1:** Relationship between the HBV-SRL expression and clinical-pathologic features.

Clinical feature	Low (*n* = 111)	High (*n* = 111)	*P* value
Age, year			0.592
Mean (SD)	51.05 (10.4)	50.68 (10.33)	
Sex, no. (%)			0.271
Male	97 (97.4)	102 (91.9)	
Female	14 (12.6)	9 (8.1)	
HBe antigen, no. (%)			0.767
Negative	78 (70.3)	80(72.1)	
Positive	33 (29.7)	31 (27.9)	
HBs antigen, no. (%)			0.695
Negative	14 (12.6)	16 (14.4)	
Positive	97 (87.4)	95 (85.6)	
Liver cirrhosis, no. (%)			0.499
No	7 (6.3)	10 (9.0)	
Yes	104 (93.7)	101 (91.0)	
Tumor size, no. (%)			0.412
≤5 cm	48 (43.2)	42 (37.8)	
>5 cm	63 (56.8)	69 (62.2)	
Tumor number, no. (%)			0.686
Single	96 (86.5)	98 (88.3)	
Multiple	15 (13.5)	13 (11.7)	
Microvascular invasion, no. (%)			0.652
Absent	82 (73.9)	79 (71.2)	
Present	29 (26.1)	32 (28.8)	
Tumor encapsulation, no. (%)			0.044
No	33 (29.7)	49 (44.1)	
Incomplete	39 (35.1)	25 (22.5)	
Complete	39 (35.1)	37 (33.3)	
Portal vein tumor thrombus, no. (%)			0.187
No	98 (88.3)	91 (82.0)	
Yes	13 (11.7)	20 (18.0)	
Ascites, no. (%)			0.075
No	102 (91.9)	108 (97.3)	
Yes	9 (8.1)	3 (2.7)	
Alpha-fetoprotein, no. (%)			0.779
Negative, ≤20 (ng/ml)	38 (34.2)	40 (36.0)	
Positive, >20 (ng/ml)	73 (65.8)	71 (64.0)	
HBV DNA, no. (%)			0.021
≤10000 (copies/ml)	72 (64.9)	55 (49.5)	
>10000 (copies/ml)	39 (35.1)	56 (50.5)	
Serum bilirubin, no. (%)			0.652
≤18.8 (*μ*mol/l)	79 (71.2)	82 (73.9)	
>18.8 (*μ*mol/l)	32 (28.8)	29 (26.1)	
Serum albumin, no. (%)			0.196
≤40 (g/l)	7 (6.3)	3 (2.7)	
>40 (g/l)	104 (93.7)	108 (97.3)	
Serum prealbumin, no. (%)			0.324
≤170 (mgl)	42 (37.8)	35 (31.5)	
>170 (mg/l)	69 (62.2)	76 (68.5)	
Alanine aminotransferase, no. (%)			0.419
≤41 (U/liter)	63 (56.8)	57 (51.4)	
>41 (U/liter)	48 (43.2)	54 (48.6)	
Aspartate aminotransferase, no. (%)			0.502
≤37 (U/liter)	52 (46.8)	57 (51.4)	
>37 (U/liter)	59 (53.2)	54 (48.6)	

*P* value <0.05 was considered to indicate statistical significance. *P* values were calculated using Fisher's exact test, except for age, which was calculated with an unpaired *t* test.

**Table 2:** Univariate analyses of factors associated with survival and recurrence for 222 HCC patients.

Factor	OS	DFS
	*P*	*P*
Age (yr)		
>60 Vs ≤ 60	0.427	0.008
Sex		
Male vs. female	0.417	0.107
HBsAg		
Positive vs. negative	0.53	0.374
HBV DNA		
>10^4^ Vs ≤ 10^4^	<0.001	<0.001
AFP (*μ*g/L)		
>20 Vs ≤ 20	0.004	0.003
ALT (U/L)		
>41 Vs ≤ 41	0.209	0.028
AST (U/L)		
>37 Vs ≤ 37	0.002	<0.001
Tumor number		
Multiple vs. single	0.009	<0.001
Liver cirrhosis		
Yes vs. no	0.156	0.598
Tumor size (cm)		
>5 Vs ≤ 5	<0.001	<0.001
Micrometastases		
Yes Vs. no	<0.001	<0.001
Tumor encapsulation		
Yes Vs. no	<0.001	<0.001
Microvascular invasion		
Yes Vs no	0.013	<0.001
HBV-SRL		
High Vs. low	0.002	0.043
NF-*κ*B2		
High Vs. low	0.001	0.041

OS, overall survival; DFS, disease-free survival; PVTT, portal vein tumor thrombus; HBsAg, hepatitis B surface antigen; HBV DNA, hepatitis B virus deoxyribonucleic acid; TNM, tumor-node metastasis; AFP, *α*-fetoprotein; NA, not adopted; NS, not significant; Low/high, HBV-SRL, and NF*κ*B levels were lower/higher than the median value.

## Data Availability

The data used to support the findings of this study are included within the article.

## Supplementary Materials

Supplementary Materials The supplementary materials for this article include 6 figures and 4 tables, and the contents are shown by the figures: Supplementary Figure 1: altered expressed lncRNAs between tumor and corresponding parenchyma tumor tissues. Supplementary Figure 2: diagram of the constructs used for the HBV-SRL-His expression. Supplementary Figure 3: expression of HBV-SRL in Hep3B cells transfected with plasmids or siRNAs. Supplementary Figure 4: expression of NF-*κ*B2 in Hep3B cells transfected with plasmids or siRNAs. Supplementary Figure 5: cell cycle analysis of tumor cells using flow cytometry. Supplementary Figure 6: expression of NF-*κ*B2 in tumor tissues and its correlation with prognosis of HCC patients with HBV infection. The contents are shown by tables: Supplementary Table 1: clinicopathologic features of 222 HCC patients with HBV. Supplementary Table 2: upregulated lncRNAs in HBV + tumors selected as candidate molecules. Supplementary Table 3: the promoters associated with HBV-SRL from Blat analysis. Supplementary Table 4: primers and siRNA sequences. Also, a document describing patients' information and some experimental methods is included.
