# Supplementary material for: The HBV Specially-Related Long Noncoding RNA HBV-SRL Involved in the Pathogenesis of Hepatocellular Carcinoma
Source: J Oncol. 2022 Jul 8;2022:9034105. doi: 10.1155/2022/9034105 (PMC9286890; doi:10.1155/2022/9034105)
Supplement: Supplementary Materials — The supplementary materials for this article include 6 figures and 4 tables, and the contents are shown by the figures: Supplementary Figure 1: altered expressed lncRNAs between tumor and corresponding parenchyma tumor tissues. Supplementary Figure 2: diagram of the constructs used for the HBV-SRL-His expression. Supplementary Figure 3: expression of HBV-SRL in Hep3B cells transfected with plasmids or siRNAs. Supplementary Figure 4: expression of NF-κB2 in Hep3B cells transfected with plasmids or siRNAs. Supplementary Figure 5: cell cycle analysis of tumor cells using flow cytometry. Supplementary Figure 6: expression of NF-κB2 in tumor tissues and its correlation with prognosis of HCC patients with HBV infection. The contents are shown by tables: Supplementary Table 1: clinicopathologic features of 222 HCC patients with HBV. Supplementary Table 2: upregulated lncRNAs in HBV + tumors selected as candidate molecules. Supplementary Table 3: the promoters associated with HBV-SRL from Blat analysis. Supplementary Table 4: primers and siRNA sequences. Also, a document describing patients' information and some experimental methods is included. [file 9034105.f1.zip › Supplementary Figure Legend (2).pdf]

Supplementary Figure. 1

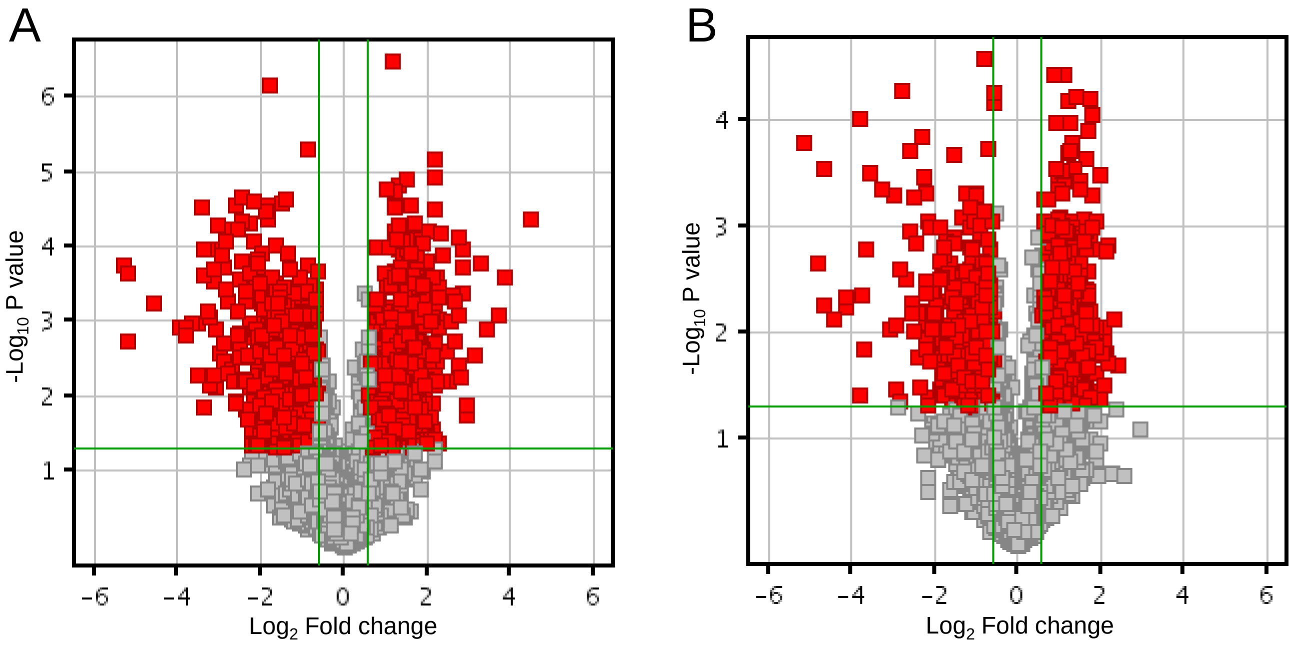

HBV$^+$ HCC T Vs PT

HBV$^-$ HCC T Vs PT

Supplementary Fig.1 Altered expressed lncRNAs between tumor and corresponding parenchyma tumor tissues.

A) Microarray analysis found differential expression of lncRNAs between tumor tissues (T) and corresponding parenchyma tumor tissues (PT) with HBV (HBV$^+$ HCC).

B) Microarray analysis found differential expression of lncRNAs between tumor tissues (T) and corresponding parenchyma tumor tissues (PT) without HBV (HBV$^-$ HCC).

Supplementary Figure. 2

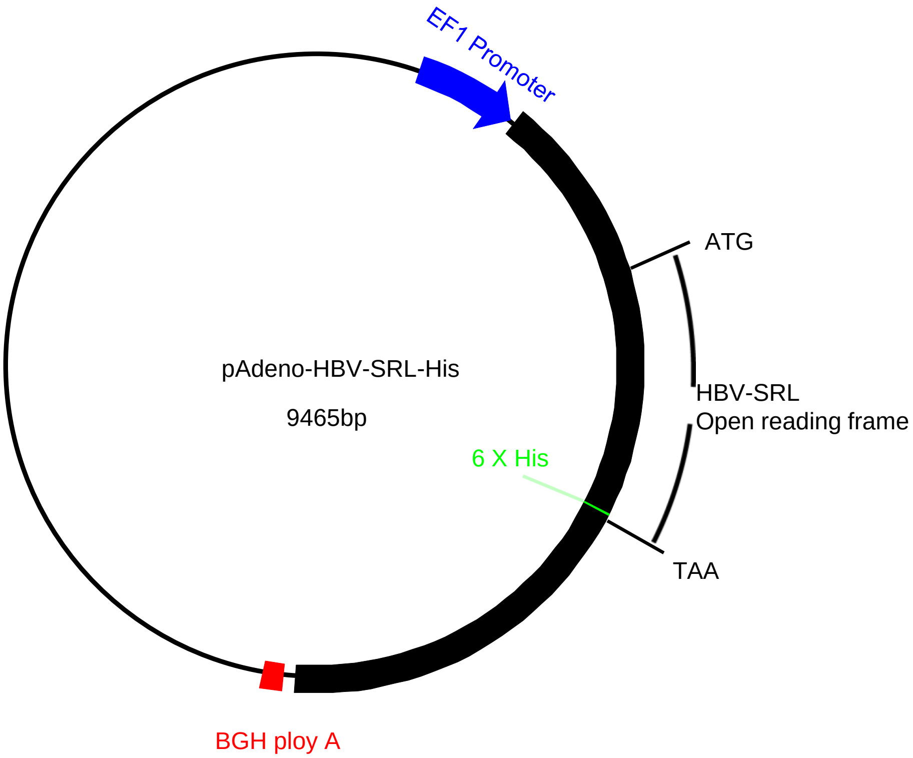

Supplementary Fig.2 Diagram of the constructs used for HBV-SRL-His expression.

Supplementary Figure. 3

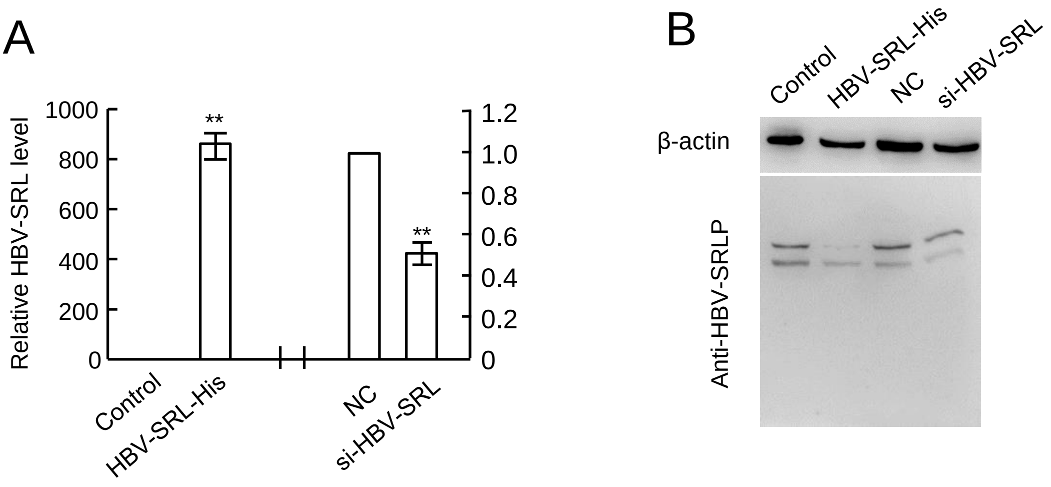

Supplementary Fig.3   Expression of HBV-SRL in Hep3B cells transfected with plasmids or siRNAs.

(A) Real-time PCR analysis of HBV-SRL expression in Hep3B cells transfected with plasmid pAdeno-His (Control), pAdeno-HBV-SRL-His (HBV-SRL-His) or siRNAs targeting HBV-SRL (si-HBV-SRL) and NC control (NC). The error bars represent the standard deviation (SD) of data obtained in at least three independent experiments. **P< 0.01

(B) Western blot analysis performed using proteins from Hep3B cells using Anti-HBV-SRLP.

Supplementary Figure 4

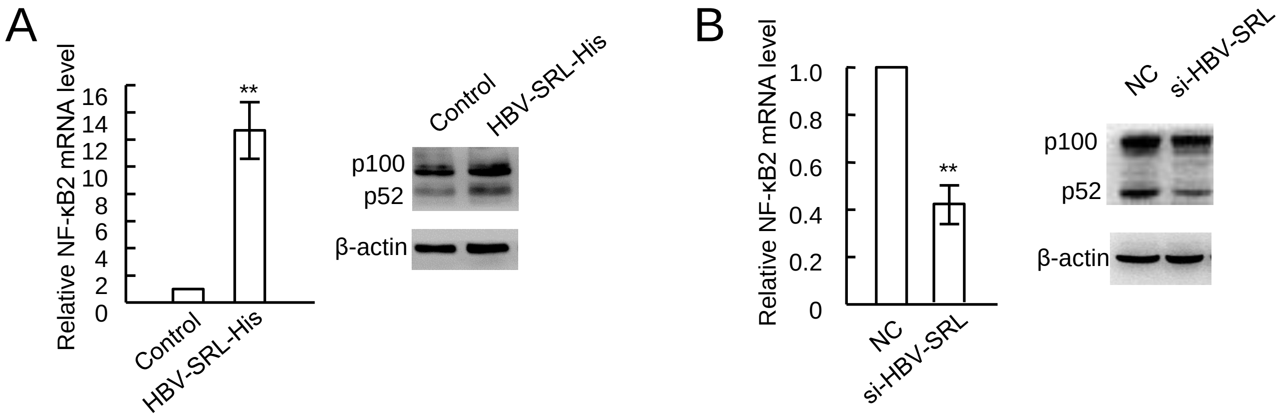

Supplementary Fig.4 Expression of NF-kB2 in Hep3B cells transfected with plasmids or siRNAs.

(A) Real-time PCR (*left*) and Western blot (*right*) analysis of NF-kB2 in Hep3B cells transfected with plasmid pAdeno-His (Control), pAdeno-HBV-SRL-His (HBV-SRL-His).

(B) Real-time PCR (*left*) and Western blot (*right*) analysis of NF-kB2 in Hep3B cells transfected with siRNAs targeting HBV-SRL (si-HBV-SRL) and NC control (NC).

The error bars represent the standard deviation (SD) of data obtained in at least three independent experiments. **P< 0.01

Supplementary Figure. 5

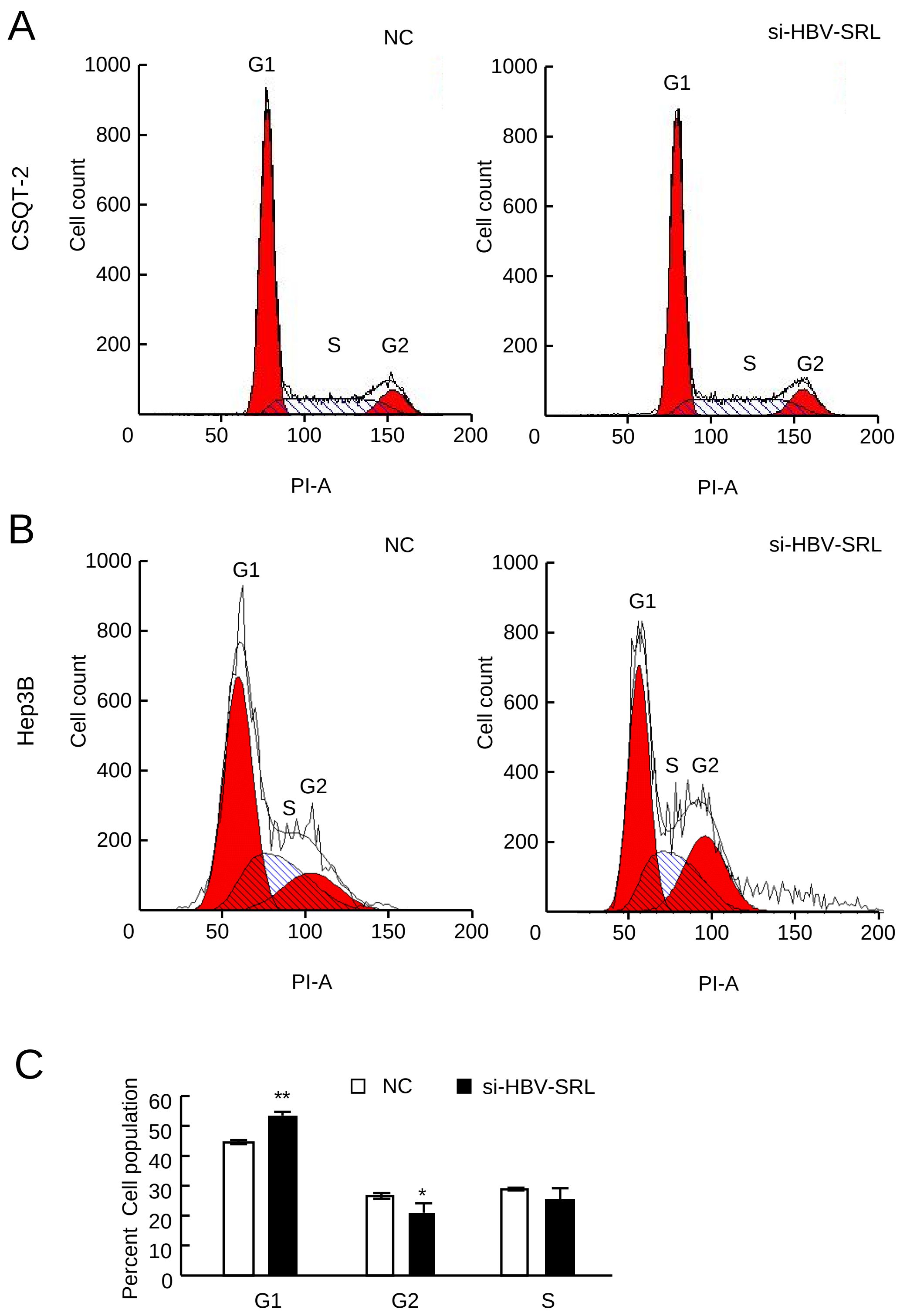

Supplementary Fig.5   Cell cycle analysis of tumor cells using Flow cytometry.

(A) Represent image of cell cycle analysis of CSQT-2 cells transfected with siRNAs targeting HBV-SRL (si-HBV-SRL) and NC control (NC) using Flow cytometry.

(B) Represent image of cell cycle analysis of Hep3B cells transfected with siRNAs targeting HBV-SRL (si-HBV-SRL) and NC control (NC) using Flow cytometry.

(C) Cell cycles of Hep3B cells transfected with siRNAs targeting HBV-SRL (si-HBV-SRL) and NC control (NC) analysed using Flow cytometry.

The error bars represent the standard deviation (SD) of data obtained in at least three independent experiments. *P< 0.05, **P< 0.01

Supplementary Figure. 6

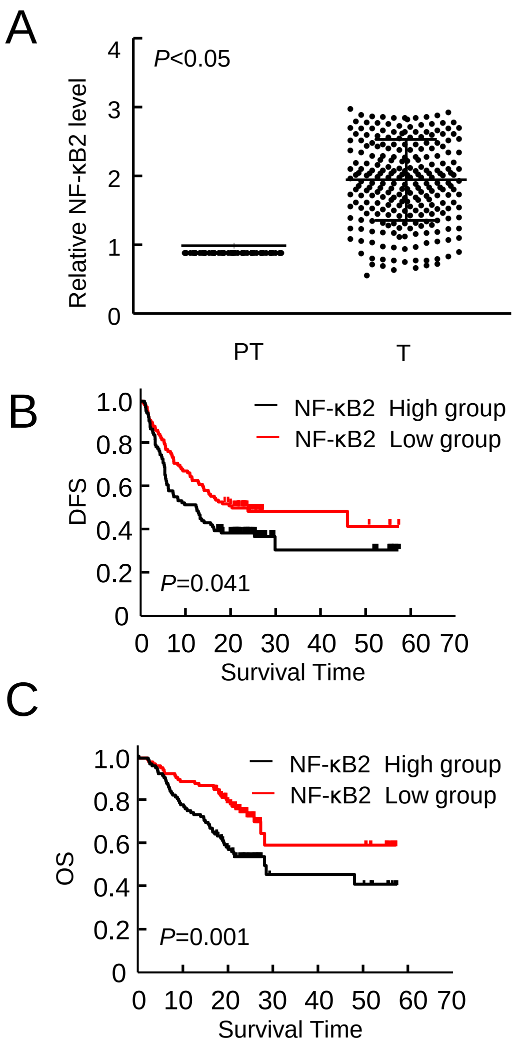

Supplementary Fig.6 Expression of NF-κB2 in tumor tissues and its Correlation with prognosis of HCC patients with HBV infection

A) Real-time PCR analysis of NF-κB2 expression in tumor tissues

(T) and corresponding parenchyma tumor tissues (PT) from 222

HCC patients with HBV.

B) Correlations of NF-κB2 with DFS of 222 HCC patients analysed by Kaplan-Meier's analyses.
