# Supplementary material for: The HBV Specially-Related Long Noncoding RNA HBV-SRL Involved in the Pathogenesis of Hepatocellular Carcinoma
Source: J Oncol. 2022 Jul 8;2022:9034105. doi: 10.1155/2022/9034105 (PMC9286890; doi:10.1155/2022/9034105)
Supplement: Supplementary Materials — The supplementary materials for this article include 6 figures and 4 tables, and the contents are shown by the figures: Supplementary Figure 1: altered expressed lncRNAs between tumor and corresponding parenchyma tumor tissues. Supplementary Figure 2: diagram of the constructs used for the HBV-SRL-His expression. Supplementary Figure 3: expression of HBV-SRL in Hep3B cells transfected with plasmids or siRNAs. Supplementary Figure 4: expression of NF-κB2 in Hep3B cells transfected with plasmids or siRNAs. Supplementary Figure 5: cell cycle analysis of tumor cells using flow cytometry. Supplementary Figure 6: expression of NF-κB2 in tumor tissues and its correlation with prognosis of HCC patients with HBV infection. The contents are shown by tables: Supplementary Table 1: clinicopathologic features of 222 HCC patients with HBV. Supplementary Table 2: upregulated lncRNAs in HBV + tumors selected as candidate molecules. Supplementary Table 3: the promoters associated with HBV-SRL from Blat analysis. Supplementary Table 4: primers and siRNA sequences. Also, a document describing patients' information and some experimental methods is included. [file 9034105.f1.zip › Supplementary material and methods (1).docx]

**Supplementary Materials and methods**

**Patients and follow-up**

Two cohorts of HCC patients who underwent radical cancer resection at the Eastern Hepatobiliary Surgery Hospital (EHBH) were included for microarray analysis and validation respectively in the present study. The cohort 1 including both HBV positive and negative HCC patients (n=5 each group) was used for mRNA microarray analysis. And the cohort 2, used for validation, included HBV positive HCC patients (n=222) who underwent radical cancer resection from June 1, 2008 to September 30, 2009 with the clinical characteristics listed in Supplementary Table 1. The study was approved by the Institutional Review Board of the EHBH.

All patients were observed until June 30, 2012 and follow-up assessments were performed every month for the first 2 years and every 3 months thereafter. Outcome measures of postoperative included disease-free survival (DFS) and overall survival (OS) rate^1^.

Hepatectomies were performed by an independent surgical team at the EHBH. Pre-operative clinical diagnosis of HCC met the diagnostic criteria of the American Association for the Study of Liver Diseases^2,3^. Snap-frozen or paraffin-embedded specimens of tumors and adjacent hepatic tissues from 222 HCC patients were obtained with informed consent. The data does not contain any information that could be used to identify the patients.

**Microarray analysis**

LncRNA and mRNA microarray analysis were performed using HCC tumor tissues from patients with (Group 1, n=5) or without HBV (Group 2, n=5) as described previously^4^.

**Cell line**

The tumor cell line CSQT-2 was established in our lab^5^. Hep3B were obtained from the American Tissue Culture Collection (ATCC). The cell lines were maintained in high-glucose DMEM (Gibco BRL, Grand Island, NY) supplemented with 10% fetal bovine serum (Gibco BRL), 100 mg/mL penicillin G, and 50 μg/mL streptomycin (Gibco BRL) at 37°C in a humidified atmosphere containing 5% CO_2_. The cell lines was authenticated using short tandem repeat profiling by Hkgene (Beijing, China)

**Real-time PCR**

Total RNA was isolated from cell lines and tissues using TRIzol reagent (Invitrogen, Carlsbad, CA) and reverse transcribed using M-MLV Reverse Transcriptase kit (Invitrogen) according to the manufacturer’s instructions. Real-time PCR was performed with the SYBR Premix Ex Taq™ (TaKaRa Bio Inc. Otsu Shiga, Japan) using the StepOne Plus Real-Time PCR system (Applied Biosystems, Foster City, CA). The primers sequences are listed in Supplementary Table 4. The gene expression levels were calculated relative to the expression of β-actin using the 2^-ΔΔCt^ method or the 2^-ΔCt^ method.

**Western Blot**

Western blot was performed as previously described ^4^. The antibody NF-ΚB2 P100/P52 (18D10) (#3017, Cell Signaling Beverly, MA) and His-Tag (#12698, Cell Signaling) was used.

**Cell cycle analysis**

CSQT-2 cells were incubated with PI/RNase Staining Buffer (BD Pharmingen) following incubation with cold 70% ethanol. Data was acquired by Flow cytometer and analysed by ModFit LT.

**Cell proliferation Measurement**

The proliferation of CSQT-2 cells transfected with siRNAs or plasmid was measured by the Cell Counting Kit-8 (CCK-8, Dojindo) assay. The absorbance at 450 nm was measured using a microplate reader (BIO-RAD), and the background absorbance was corrected using the CCK-8 solution without cells.

**Luciferase reporter assay**

CSQT-2 cells (5 × 10^4^) seeded in 24-well plates were transfected with the Firefly luciferase reporter plasmid (500ng, pGL3-*NF-κB2* promoter-wild type or pGL3-*NF-κB2* promoter-mutant), the Renilla luciferase control plasmid (50ng, pRL-TK) and pAdeno-HBV-SRL-His (500ng) plasmid. 24 hours after transfection, the assays were performed using the Dual Luciferase Reporter Assay System (Promega).

**References**

1. Liu S, Guo W, Shi J, et al: MicroRNA-135a contributes to the development of portal vein tumor thrombus by promoting metastasis in hepatocellular carcinoma. J Hepatol 56:389-96, 2012

2. Bruix J, Sherman M: Management of hepatocellular carcinoma. Hepatology 42:1208-36, 2005

3. Semmler G, Meyer EL, Kozbial K, et al: HCC risk stratification after cure of hepatitis C in patients with compensated advanced chronic liver disease. J Hepatol 76:812-821, 2022

4. Guo W, Liu S, Cheng Y, et al: ICAM-1-Related Noncoding RNA in Cancer Stem Cells Maintains ICAM-1 Expression in Hepatocellular Carcinoma. Clin Cancer Res, 2015

5. Wang T, Hu HS, Feng YX, et al: Characterisation of a novel cell line (CSQT-2) with high metastatic activity derived from portal vein tumour thrombus of hepatocellular carcinoma. Br J Cancer 102:1618-26, 2010
