# Supplementary material for: The HBV Specially-Related Long Noncoding RNA HBV-SRL Involved in the Pathogenesis of Hepatocellular Carcinoma
Source: J Oncol. 2022 Jul 8;2022:9034105. doi: 10.1155/2022/9034105 (PMC9286890; doi:10.1155/2022/9034105)
Supplement: Supplementary Materials — The supplementary materials for this article include 6 figures and 4 tables, and the contents are shown by the figures: Supplementary Figure 1: altered expressed lncRNAs between tumor and corresponding parenchyma tumor tissues. Supplementary Figure 2: diagram of the constructs used for the HBV-SRL-His expression. Supplementary Figure 3: expression of HBV-SRL in Hep3B cells transfected with plasmids or siRNAs. Supplementary Figure 4: expression of NF-κB2 in Hep3B cells transfected with plasmids or siRNAs. Supplementary Figure 5: cell cycle analysis of tumor cells using flow cytometry. Supplementary Figure 6: expression of NF-κB2 in tumor tissues and its correlation with prognosis of HCC patients with HBV infection. The contents are shown by tables: Supplementary Table 1: clinicopathologic features of 222 HCC patients with HBV. Supplementary Table 2: upregulated lncRNAs in HBV + tumors selected as candidate molecules. Supplementary Table 3: the promoters associated with HBV-SRL from Blat analysis. Supplementary Table 4: primers and siRNA sequences. Also, a document describing patients' information and some experimental methods is included. [file 9034105.f1.zip › Supplementary Table (1).doc]

**Supplementary Table 1. Clinicopathologic features of 222 HCC patients with HBV**

| Clinical feature |  |  |
| --- | --- | --- |
| Age — Year | | |
| Mean(SD) | 50.87(10.34) |  |
| Sex — No. (%) | | |
| Male | 199 (97.4) |  |
| Female | 23 (12.6) |  |
| HBe antigen — No. (%) | | |
| Negative | 158 (70.3) |  |
| Positive | 64 (29.7) |  |
| HBs antigen — No. (%) | | |
| Negative | 30 (12.6) |  |
| Positive | 192 (87.4) |  |
| Liver cirrhosis — No. (%) | | |
| No | 17 (6.3) |  |
| Yes | 205(93.7) |  |
| Tumor size — No. (%) | | |
| ≤ 5 cm | 90 (43.2) |  |
| > 5 cm | 132(56.8) |  |
| Tumor number — No. (%) | | |
| Single | 194(86.5) |  |
| Multiple | 28 (13.5) |  |
| Microvascular invasion — No. (%) | | |
| Absent | 161 (73.9) |  |
| Present | 61 (26.1) |  |
| Tumor encapsulation — No. (%) | | |
| No | 82 (29.7) |  |
| Incomplete | 64 (35.1) |  |
| Complete | 76 (35.1) |  |
| Portal vein tumor thrombus — No. (%) | | |
| No | 189 (88.3) |  |
| Yes | 33 (11.7) |  |
| Ascites — No. (%) | | |
| No | 210 (91.9) |  |
| Yes | 12 (8.1) |  |
| Alpha-fetoprotein — No. (%) | | |
| Negative, ≤20 ng/ml | 78 (34.2) |  |
| Positive, >20 ng/ml | 144 (65.8) |  |
| HBV DNA — No. (%) | | |
| ≤10000 copies/ml | 127 (64.9) |  |
| >10000 copies/ml | 95 (35.1) |  |
| Serum bilirubin — No. (%) | | |
| ≤18.8 μmol/l | 161 (71.2) |  |
| >18.8 μmol/l | 61 (28.8) |  |
| Serum albumin — No. (%) | | |
| ≤40 g/l | 10 (6.3) |  |
| >40 g/l | 212 (93.7) |  |
| Serum prealbumin — No. (%) | | |
| ≤170 mgl | 77 (37.8) |  |
| >170 mg/l | 145 (62.2) |  |
| Alanine aminotransferase — No. (%) | | |
| ≤41 U/liter | 120 (56.8) |  |
| >41 U/liter | 102 (43.2) |  |
| Aspartate aminotransferase — No. (%) | | |
| ≤37 U/liter | 109 (46.8) |  |
| >37 U/liter | 113 (53.2) |  |

*P* value <0.05 was considered to indicate statistical significance. *P* values were calculated using Fisher’s exact test, except for age, which was calculated with an unpaired t-test.

**Supplementary Table 2. Up-regulated lncRNAs in HBV^+^ Tumors Selected as candidate molecules**

| Name | HBV^+^T Vs HBV^+^PT | | HBV^+^ T Vs HBV^-^T | | Chromosome (Strand)  Start-End | Source |
| --- | --- | --- | --- | --- | --- | --- |
|  | P | Fold change | P | Fold change |  |  |
| AK128595 | 0.00 | 22.39 | 0.02 | 4.94 | chr16(-)  4430310-4782833 | mRNA |
| AX800134 | 0.00 | 14.46 | 0.00 | 3.73 | chr15(-)  83759460-83760087 | mRNA |
| uc003olt | 0.00 | 7.24 | 0.01 | 5.48 | chr6(+)  36206239-36215820 | UCSC |
| NR_002935 | 0.00 | 7.21 | 0.00 | 2.52 | chr7(+)  151764912-151771033 | RefNR |

*P, P* value < 0.05 was considered to indicate statistical significance; HBV^+^T, HBV positive HCC tumors; HBV^+^PT, HBV positive parenchyma tumor tissues; HBV^-^T, HBV negative HCC tumors;

Supplementary Table 3. The promoters associated with HBV-SRL from Blat analysis

| Gene Name | Seq Name | Length(bp) | | | |
| --- | --- | --- | --- | --- | --- |
|  |  | Total | Match | Mismatch | Strand |
| RELB | NM_006509_promoter | 1500 | 324 | 25 | + |
| NFKB2 transcript variant 1 | NM_001077494_promoter | 1500 | 80 | 3 | + |
| NFKB2 transcript variant 3 | NM_001077493_promoter | 1500 | 80 | 3 | + |
| NFKB2 transcript variant 1 | NM_001077494_promoter | 1500 | 238 | 23 | - |
| NFKB2 transcript variant 3 | NM_001077493_promoter | 1500 | 238 | 23 | - |

Strand: -, complementary; +, similarity.

Supplementary Table 4. Primers and siRNA Sequences

| **Name** | | **Sequence** |
| --- | --- | --- |
| HBV-SRL | Forward | 5ˊ- TCCTCTTCACCTCATCAC -3′ |
|  | Reverse | 5ˊ- CCTACACTTAGCGTTCAG -3′ |
| NF-KB2 | Forward | 5ˊ- ATTGAGGTTCGGTTCTATGAGG -3′ |
|  | Reverse | 5ˊ- AACACTGTTACAGGCCGCTCA -3′ |
| β-actin | Forward | 5ˊ- GCACCACACCTTCTACAATGAG -3′ |
|  | Reverse | 5ˊ- ACAGCCTGGATGGCTACGT -3′ |
| *NF-KB2*  promoter | Forward | 5ˊ- ATGGCACCTGCTAAGGAGAAAC-3′ |
|  | Reverse | 5ˊ- GCTCCGTCTCATCAGCCTCAA-3′ |
| HBV-SRL siRNA sequence | |  |
| siRNA1 | Sense | 5ˊ- GGUAGUUAGUCCUUAUUAATT -3′ |
|  | Anti-sense | 5ˊ- UUAAUAAGGACUAACUACCTT -3′ |
| siRNA2 | Sense | 5ˊ- GGCAGGAGAAUCACUUGAATT -3′ |
|  | Anti-sense | 5ˊ- UUCAAGUGAUUCUCCUGCCTT -3′ |
| siRNA3 | Sense | 5ˊ- GUGAAACCCUGUCUCUACUTT  -3′ |
|  | Anti-sense | 5ˊ- AGUAGAGACAGGGUUUCACTT  -3′ |
| NC | Sense | 5ˊ-UUCUCCGAACGUGUCACGUTT -3′ |
|  | Anti-sense | 5ˊ-ACGUGACACGUUCGGAGAATT -3′ |

HBV-SRL, long noncoding RNA; siRNA-1/2/3, small interfering RNA (siRNA) sequence targeting HBV-SRL; NC, negative control;
